# Gene-Based Tests of a Genome-Wide Association Study Dataset Highlight Novel Multiple Sclerosis Risk Genes

**DOI:** 10.3389/fnins.2021.614528

**Published:** 2021-05-11

**Authors:** He Li, Xiaodan Hou, Yan Liang, Fang Xu, Xiyue Zhang, Pan Cui, Gebeili Xing, Xuejiao Wang, Wei Jiang

**Affiliations:** ^1^Department of Neurology, Tianjin Neurological Institute, Tianjin Medical University General Hospital, Tianjin, China; ^2^Department of Neurology, Inner Mongolia People’s Hospital, Hohhot, China; ^3^Department of Neurology, Datong Third People’s Hospital, Datong, China

**Keywords:** multiple sclerosis, gene sets, gene differential expression, genome-wide association study, gene expression omnibus

## Abstract

Multiple sclerosis (MS) is an autoimmune disorder influenced by genetic and environmental factors. Many studies have provided insights into genetic factors’ contribution to MS via large-scale genome-wide association study (GWAS) datasets. However, genetic variants identified to date do not adequately explain genetic risks for MS. This study hypothesized that novel MS risk genes could be identified by analyzing the MS-GWAS dataset using gene-based tests. We analyzed a GWAS dataset consisting of 9,772 MS cases and 17,376 healthy controls of European descent. We performed gene-based tests of 464,357 autosomal single nucleotide polymorphisms (SNPs) using two methods (PLINK and VEGAS2) and identified 28 shared genes satisfied *p*-value < 4.56 × 10^–6^. In further gene expression analysis, ten of the 28 genes were significantly differentially expressed in the MS case-control gene expression omnibus (GEO) database. GALC and HLA-DOB showed the most prominent differences in gene expression (two- and three-fold, respectively) between MS patients and healthy controls. In conclusion, our results reveal more information about MS hereditary characteristics and provide a basis for further studies.

## Introduction

Multiple sclerosis (MS) is a neurodegenerative, inflammatory, demyelinating, and autoimmune disease of the central nervous system (CNS) that causes widespread tissue lesions and dysfunction (McFarland and Martin, 2007). As a result, the communication between neurons is disrupted, as demonstrated by extensive signs and symptoms (Filippi and Rocca, 2005). The etiology of MS involves genetic as well as environmental factors (Canto and Oksenberg, 2018). However, genetic factors have more vital roles in MS pathogenesis than environmental ones (Dendrou et al., 2015). Genome-wide association studies (GWAS) have recently revealed genetic risk factors for MS (International Multiple Sclerosis Genetics Consortium [IMSGC], 2019) and confirmed 233 loci, including 201 non-MHC and 32 MHC variants.

Although GWAS have helped comprehend the genetic basis of human diseases, there are still some limitations, including the unprecedented potential to produce false-positive results, lack of information on gene function, insufficient sample size, lack of well-defined case and control groups, and insensitivity to rare, and structural variants (Pearson and Manolio, 2008). Gene-based tests have been considered as a complement to GWAS (Wang et al., 2011). First, they can be more potent than individual SNP-based GWAS when multiple causal interactions affect the phenotype of interest (Ma et al., 2013). Second, gene-based tests combine all SNPs into a single test and correct linkage disequilibrium (LD) (Ma et al., 2013). Also, gene-based approaches reduce false positives that arise from multiple testing in GWAS (Liu et al., 2010). Finally, gene-based analysis prioritizes GWAS results and determines the association between diseases and functional genetic analysis (e.g., a shared biological pathway) (Wang et al., 2011).

Gene-based tests of large-scale MS-GWAS datasets provide strong support for the identification of novel MS susceptibility loci. This study performed gene-based tests of an MS-GWAS dataset that comprised 9,772 MS cases along with 17,376 control subjects using two methods. We also analyzed two MS case-control gene expression datasets to investigate further the differential expression of MS risk genes identified by both methods.

## Materials and Methods

### MS-GWAS Dataset

We used a large-scale MS-GWAS dataset from the International Multiple Sclerosis Genetics Consortium (IMSGC), which consisted of 9,772 MS cases along with 17,376 control cases of European descent collected by 23 research teams from 15 countries (Sawcer et al., 2011). After subjecting the data to the novel SNP-based quality control procedure, 464,357 autosomal SNPs were available for further analysis (Sawcer et al., 2011).

### Gene-Based Testing of MS-GWAS Dataset Using PLINK

Based on the association analysis of the MS-GWAS dataset, a gene-based test was carried out with the PLINK software (SET SCREEN TEST)^1^ (Purcell et al., 2007). The combined chi-square statistic for each SNP of a given gene was calculated using the following formula:

(1) $$x_{0}^{2}=-2\,\sum_{i=1}^{N}\ln\left(p_{i}\right)$$

N represents the number of markers (tests), and *p*_*i*_ (i = 1 … N) are the corresponding *p*-values under the null hypothesis. x_0_^2^ follows a chi-squared distribution with 2N degrees of freedom (df). If the tests (for each SNP) are not independent, the statistic x_0_^2^ has a mean m = 2N and variance (σ^2^),

$$\sigma^{2}=4\,N+\sum_{i=1}^{N-1}\sum_{j=I+1}^{N}c\,o\,v\,\left(-2\,\ln\left(p_{i}\right),-2\,\ln\left(p_{j}\right)\right)$$

where *p*_*i*_ and *p*_*j*_ (i, j = 1, …, N) are the *p*-values for each test, and the covariance (cov) is calculated as:

$$c\,o\,v\,\left(-2\,\ln\left(p_{i}\right),-2\,\ln\left(p_{j}\right)\right)=p_{i\,j}\,\left(3.25+0.75\,p_{i\,j}\right)$$

The non-negative correlation coefficients p_*ij*_ between the two variables were approximated by the correlation between two SNPs i and j. The overall significance of multiple non-independent tests is determined using the formula:

$$x^{2}=-2\sum_{i=1}^{N}\ln{\left(p_{i}\right)}^{*}4N/\sigma^{2}$$

where x^2^ follows the central chi-squared distribution with 8*N*^2^/σ^2^ df. This method was applied to analyze the MS-GWAS dataset using the LD information from the HapMap CEU population. The set screen test integrated all SNPs’ *p*-values using an approximate Fisher’s test, an asymptotically optimal method to obtain the overall significance. Under the same null hypothesis, a set of *p*-values determined by independent tests were collected and calculated (i.e., each SNP that was not related to the disease).

### Gene-Based Testing of MS-GWAS Dataset Using VEGAS2

We also used the VEGAS2 software to perform a gene-based test (Liu et al., 2010; Mishra and Macgregor, 2015). In VEGAS2, there are five options regarding gene boundaries for the SNP option: SNPs within 0kbloc, 10kbloc, 20kbloc, 50kbloc, or 0kbldbin (Liu et al., 2010). The n SNPs’ *p*-values are first converted to upper tail χ^2^ statistics with one df for each gene definition. If SNPs are in the linkage equilibrium, VEGAS2 calculates a gene-based test statistic with a χ^2^ distribution with n df under the null hypothesis. This software can also produce more relevant SNPs where the top SNP is in high LD (Mishra and Macgregor, 2015). We selected SNPs within a gene and any SNPs outside of the gene with *r*^2^ > 0.8 for SNPs within the gene (0kbldbin) in order to decrease the specificity of the result for a given gene (Mishra and Macgregor, 2015).

### MS Case-Control Expression Analyses

We further analyzed the differential expression of shared MS risk genes from the NCBI gene expression omnibus (GEO) database^2^. Our inclusion criteria were human tissue samples, expression profiling by the array, case-control study and sample size ≥ 20. Two GEO datasets were selected for our study, GSE21942 and GSE43591 (Kemppinen et al., 2011; Jernås et al., 2013). In GEO series GSE21942, researchers recruited 12 MS female patients and 15 unrelated female controls, and all of them were Caucasian (Kemppinen et al., 2011). Peripheral blood mononuclear cells (PBMCs) isolated from whole blood were tested on the Affymetrix Gene Chip Human Genome U133 Plus 2.0 Array (Kemppinen et al., 2011). In GEO series GSE43591, ten relapsing-remitting Caucasian MS patients and 10 matched healthy controls were recruited (Jernås et al., 2013). Researchers isolated PBMCs from whole blood and sorted T cells with CD3+ positive magnetic beads. The transcriptional level was tested by Human Genome HG-U133 plus 2.0 arrays (Jernås et al., 2013).

We utilized GEO2R provided by the GEO website (GEO query along with limma R packages) to compute differential expression between case-control samples (Barrett et al., 2013). Then the transcript with the smallest *p*-value was selected among multiple transcript probes. After multiple testing correction (Benjamini-Hochberg method), the gene expression (transcript probes) with an adjusted *p*-value of <0.05 were considered to be significant.

### MS Patients and Healthy Controls

Five patients diagnosed with relapsing-remitting MS as per the McDonald Criteria of MS were enrolled from Tianjin Medical University General Hospital. The exclusion criteria were as follows: (1) co-presence of other CNS disorders; (2) diagnosis of tumors, recent infection, or systemic hematologic diseases; (3) concomitant use of antineoplastic or immune-modulating therapies before blood sampling. Five healthy volunteers were also enrolled in this study as the control group. Tianjin Medical University General Hospital’s Ethics Committee approved this study, and informed consent was obtained from each participant.

### Cell Isolation and FACS Sorting

Peripheral blood samples were obtained from all MS patients at the acute phase of the disease as well as healthy volunteers. PBMCs were isolated from blood samples using Lymphocyte Separation Medium (Solarbio) followed by Ficoll density gradient centrifugation at 2,000 × *g* for 20 min. A single-cell suspension was then prepared for FACS sorting. The following antibodies labeled with FITC, PE, and APC were used in this experiment: human reactive CD3, CD4, and CD19 (BioLegend). FACS sorting was conducted using the FACSAria Cell Sorter (BD Biosciences).

### Real-Time Quantitative PCR (RT-PCR)

TRIzol reagent (Invitrogen) was employed to isolate total RNA from sorted B lymphocytes according to the manual. The concentration of total RNA was quantified using a NanoDrop 1000 (Thermo Fisher Scientific). The total RNA was converted to cDNA with a *Trans*-Script First-Strand cDNA Synthesis SuperMix Kit (TransGen Biotech). The gene amplification was performed using the FastStart Universal SYBR Green Master (Roche). qPCR was run on the Opticon 2 Real-Time PCR Detection System (Bio-Rad). The primers used in this experiment were: HLA-DOB, F: CAGCTAAGGGCTCAGAAAGGAT and R: CTACTCATCACTACTTCAGGCTCCA; β-actin, F: AGCACAATGAAGATCAAGATCAT and R: ACTCGTCA TACTCCTGCTTGC.

## Results

### Gene-Based Tests of MS-GWAS Dataset

A total of 464,357 SNPs were mapped to 14,187 and 14,811 genes using PLINK and VEGAS2, respectively. There were 10,956 shared genes identified by both PLINK and VEGAS2. The Bonferroni correction showed that 28 genes had a *p*-value of <4.56 × 10^–6^ (*p* = 0.05/10956): AHI1, CD58, CDSN, CLEC16A, COL11A2, DPH5, EVI5, EXTL2, FCRL3, HLA-DOA, HLA-DOB, IL2RA, IQCB1, MICB, MMEL1, NFKBIL1, PSMB8, PSMB9, PSORS1C1, PSORS1C2, TAP1, TAP2, TMEM39A, TNFRSF1A, TNFSF14, DKKL1, GALC, and GFI1 (Table 1).

**TABLE 1:** Comparison of significant shared gene sets in MS produced with PLINK and VEGAS2.

Gene set	Position	PLINK			VEGAS2	
		NSNP	KB	*p*-value	NSNP	*p*-value
AHI1	chr6:135648258.135814841	15	166.6	7.20E-09	15	1.00E-06
CD58	chr1:116883333.116902480	2	19.15	2.53E-08	2	1.00E-06
CDSN	chr6:31191792.31195913	3	4.121	0	3	1.00E-06
CLEC16A	chr16:10949695.11183414	55	233.7	1.62E-10	55	1.00E-06
COL11A2	chr6:33244123.33266876	9	22.75	1.31E-10	9	1.00E-06
DPH5	chr1:101245330.101258649	3	13.32	1.84E-09	3	1.00E-06
EVI5	chr1:92753827.92991364	15	237.5	1.18E-07	16	1.00E-06
EXTL2	chr1:101122894.101132803	3	9.909	2.48E-10	5	1.00E-06
FCRL3	chr1:155914722.155935902	4	21.18	8.13E-07	4	1.00E-06
HLA-DOA	chr6:33080865.33085293	9	4.428	7.66E-07	9	1.00E-06
HLA-DOB	chr6:32888702.32892598	6	3.896	0	6	1.00E-06
IL2RA	chr10:6102114.6141719	13	39.6	3.95E-07	14	1.00E-06
IQCB1	chr3:122983760.123026267	8	42.51	1.64E-07	8	1.00E-06
MICB	chr6:31580699.31584833	2	4.134	0	4	1.00E-06
MMEL1	chr1:2516606.2543484	4	26.88	4.59E-13	4	1.00E-06
TAP1	chr6:32921257.32927843	6	6.586	1.89E-09	6	1.00E-06
TMEM39A	chr3:120633526.120657228	5	23.7	1.44E-06	6	1.00E-06
TNFRSF1A	chr12:6310270.6319376	6	9.106	3.56E-07	6	1.00E-06
TNFSF14	chr19:6616020.6619972	2	3.952	1.27E-06	3	1.00E-06
DKKL1	chr19:54559725.54570008	3	10.28	2.16E-07	3	2.00E-06
GALC	chr14:87477641.87516629	10	38.99	1.65E-06	10	2.00E-06
GFI1	chr1:92713438.92717582	2	4.144	2.23E-06	2	2.00E-06
NFKBIL1	chr6:31623778.31633427	3	9.649	0	3	1.00E-06
PSMB8	chr6:32917826.32919607	3	1.781	0	3	1.00E-06
PSMB9	chr6:32930836.32933326	3	2.49	0	3	1.00E-06
PSORS1C1	chr6:31191792.31215712	14	23.92	0	14	1.00E-06
PSORS1C2	chr6:31213392.31215066	5	1.674	0	5	1.00E-06
TAP2	chr6:32903010.32912912	18	9.902	0	18	1.00E-06

*NSNP, number of single nucleotide polymorphisms. In the VEGAS2, the *p*-value from 1.00E-06 to 0 was interpreted as *p* < 10E-06.*

### MS Case-Control Expression Analyses

Ten of the 28 genes were differentially expressed in at least one serie of MS GEO datasets after Benjamini-Hochberg correction test (adjusted *p*-value of < 0.05): AHI1 (ID 221569_at, adjusted *p* = 1.57E-02), DPH5 (ID 219590_x_at, adjusted *p* = 3.30E-02), HLA-DOA (ID 226878_at, adjusted *p* = 1.39E-02), HLA-DOB (ID 205671_s_at, adjusted *p* = 1.06E-04), TNFRSF1A (ID 207643_s_at, adjusted *p* = 3.58E-05), TMEM39A (ID 218615_s_at, adjusted *p* = 9.74E-03), TNFSF14 (ID 207907_at, adjusted *p* = 6.54E-03), GALC (ID 211810_s_at, adjusted *p* = 7.77E-04), PSMB8 (ID 209040_s_at, adjusted *p* = 2.72E-02), and TAP2 (ID 204770_at, adjusted *p* = 1.93E-02) in PBMCs; TNFRSF1A (ID 207643_s_at, adjusted *p* = 2.55E-03), TNFSF14 (ID 207907_at, adjusted *p* = 1.68E-02), and TNFSF14 (ID 207907_at, adjusted *p* = 3.56E-02) in peripheral blood T cells (PBTCs). The logFc of GALC (ID 211810_s_at, adjusted *p* = 7.77E-04) was -1.21, indicating that the expression of GALC in MS cases was 2.3-fold lower (downregulated) than that in healthy controls. By contrast, the logFc of HLA-DOB (ID 205671_s_at, adjusted *p* = 1.06E-04) was 1.57, indicating that the expression of HLA-DOB in MS cases was 3.0-fold higher (upregulated) in MS cases in contrast with control subjects (Table 2).

**TABLE 2:** Significant expression of common genes in PLINK and VEGAS2 analyses of MS.

Gene symbol	PBMCs (GSE21942)				PBTCs (GSE43591)			
	Probe ID	Adj *p-*value	*p-*value	logFc	Probe ID	Adj *p-*value	*p-*value	logFc
AHI1	221569_at	1.57E-02	1.74E-03	−5.05E-01	244699_at	3.92E-01	7.69E-02	8.27E-02
CD58	222061_at	1.17E-01	2.71E-02	−3.00E-01	222061_at	1.44E-01	1.13E-02	−1.67E-01
CDSN	206193_s_at	1.85E-01	5.34E-02	−1.09E-01	206193_s_at	6.61E-01	2.46E-01	3.94E-02
CLEC16A	231221_at	4.83E-01	2.69E-01	−7.93E-02	212786_at	4.33E-01	9.39E-02	−1.06E-01
COL11A2	216993_s_at	1.91E-01	5.62E-02	1.32E-01	213870_at	5.59E-01	1.64E-01	6.85E-02
DKKL1	220284_at	7.46E-01	5.82E-01	−3.62E-02	220284_at	8.55E-01	5.41E-01	−2.86E-02
DPH5	219590_x_at	3.30E-02	4.62E-03	2.44E-01	222360_at	6.86E-01	2.73E-01	8.58E-02
EVI5	208298_at	4.30E-01	2.20E-01	1.81E-02	209717_at	1.71E-01	1.55E-02	9.28E-02
EXTL2	209537_at	5.74E-02	9.85E-03	−4.79E-01	209537_at	9.79E-01	9.06E-01	−6.33E-03
FCRL3	231093_at	2.36E-01	7.80E-02	4.12E-01	231093_at	6.71E-01	2.57E-01	−2.54E-01
GALC	211810_s_at	7.77E-04	3.31E-05	−**1.21**	204417_at	9.20E-01	7.01E-01	−4.98E-02
GFI1	206589_at	7.79E-01	6.28E-01	8.90E-02	206589_at	1.43E-01	1.12E-02	−3.77E-01
HLA-DOA	226878_at	1.39E-02	1.47E-03	6.25E-01	226878_at	2.70E-01	3.65E-02	−1.51E-01
HLA-DOB	205671_s_at	1.06E-04	2.22E-06	**1.57**	1554984_a_at	6.70E-01	2.55E-01	5.44E-02
IL2RA	206341_at	3.36E-01	1.43E-01	1.84E-01	211269_s_at	1.81E-01	1.74E-02	1.70E-01
IQCB1	211707_s_at	7.91E-01	6.45E-01	3.46E-02	211707_s_at	2.98E-01	4.41E-02	1.57E-01
MMEL1	1552930_at	3.86E-01	1.81E-01	1.31E-01	1552930_at	9.51E-01	8.03E-01	1.67E-02
MICB	205905_s_at	4.01E-01	1.93E-01	−1.43E-01	205905_s_at	1.00E-01	5.93E-03	−1.99E-01
NFKBIL1	209973_at	5.72E-01	3.63E-01	-5.42E-02	209973_at	8.94E-01	6.31E-01	−2.17E-02
PSMB8	209040_s_at	2.72E-02	3.57E-03	−2.92E-01	209040_s_at	7.65E-01	3.71E-01	−9.26E-02
PSMB9	204279_at	1.00E-01	2.16E-02	−2.69E-01	204279_at	8.78E-01	5.92E-01	−7.76E-02
PSORS1C1	220362_at	6.55E-01	4.62E-01	1.99E-02	220362_at	9.08E-01	6.65E-01	−1.88E-02
PSORS1C2	220635_at	3.38E-01	1.45E-01	1.94E-02	220635_at	8.16E-01	4.59E-01	4.60E-02
TAP1	202307_s_at	8.33E-01	7.09E-01	−4.37E-02	202307_s_at	4.66E-01	1.09E-01	−3.09E-01
TAP2	204770_at	1.93E-02	2.27E-03	4.60E-01	204770_at	7.63E-01	3.69E-01	−1.47E-01
TNFRSF1A	207643_s_at	3.58E-05	4.73E-07	-6.25E-01	207643_s_at	2.55E-03	6.18E-06	−5.85E-01
TMEM39A	218615_s_at	9.74E-03	9.34E-04	-4.58E-01	218615_s_at	1.17E-01	7.78E-03	−1.40E-01
TNFSF14	207907_at	6.54E-03	5.54E-04	−8.60E-01	207907_at	1.68E-02	2.48E-04	−4.27E-01

*PBMCs, peripheral blood mononuclear cells; PBTCs, peripheral blood T cells; logFc, log2-fold change; probe ID, transcript probes. The bold values of gene expression mean —logFc— > 1.*

### The mRNA Expression of HLA-DOB Was Different Between MS Patients and Healthy Subjects

**FIGURE 1:**
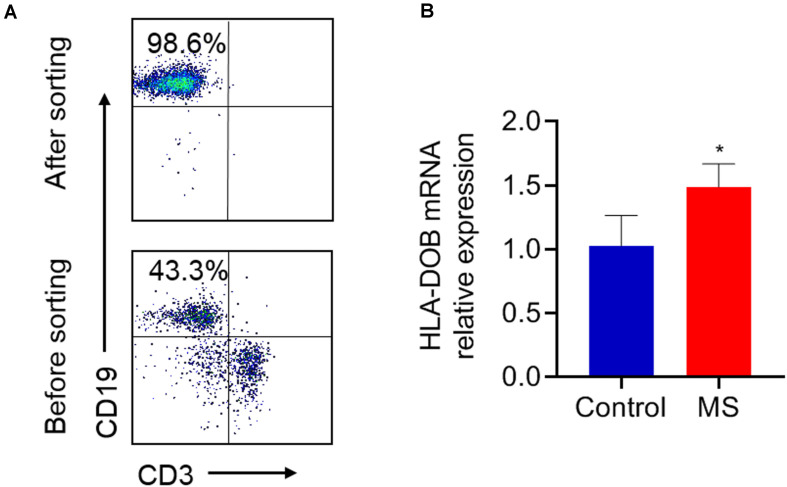
The expression levels of HLA-DOB gene in MS patients and healthy controls. **(A)** B lymphocytes (purity ≈98%) were collected from the PBMCs of MS patients, as well as healthy controls and sorted by FACS. **(B)** RT-PCR showed that the mRNA expression of HLA-DOB in MS patients was higher than that in healthy controls. Data are presented as mean ± SEM; *p < 0.05 in contrast with the control group; *n* = 5 per group.

B lymphocytes were collected from the PBMCs of MS patients, as well as healthy controls and then sorted by FACS (Figure 1A). HLA-DOB’s mRNA expression was upregulated in MS patients compared to healthy controls (Figure 1B; *p* < 0.05).

## Discussion

Although more than 200 MS susceptibility loci have been identified, a better understanding of MS genetic risk factors is still needed. The gene-based tests are widely used complemental methods to the traditional SNP-GWAS. Herein, we selected an MS-GWAS dataset from IMSGC that contained 464,357 autosomal SNPs. We performed two gene-based tests of the MS-GWAS dataset using PLINK and VEGAS2. In total, we identified 28 significant shared genes with *p*-value of <4.56 × 10^–6^ using the Bonferroni correction. Ten of them had significant differential expression at least one series of MS GEO datasets with an adjusted *p*-value of <0.05.

In our finding, ten shared MS risk genes have significantly differential expression. Among these genes, GALC and HLA-DOB showed two to threefold upregulation/downregulation in MS patients compared to controls. Moreover, TNFRSF1A and TNFSF14 genes both had significantly differential expression in PBMCs and PBTCs transcriptional data.

In gene expression analyses, GALC expressed differentially with a logFc of 1.21. Nearly 2.3-fold upregulation of GALC expression in MS patients suggests that it plays a vital role in MS’s pathogenesis. The GALC gene provides instructions for encoding galactosylceramidase, a lysosomal enzyme that hydrolyzes galactolipids, such as galactosylceramide and psychosine (Lee et al., 2007). GALC SNPs are now known to be a significant cause of Krabbe disease (KD) (Wenger et al., 2000). KD is occasionally misdiagnosed as MS due to shared pathological features of myelin lesions (Wenger et al., 1997). The GALC gene is considered a susceptibility locus for MS. By using custom ImmunoChip arrays and replication analysis of MS-GWAS datasets, IMSGC identified 48 novel risk variants for MS, including GALC (rs74796499), with a joint *p*-value of 2.4E-20 (Beecham et al., 2013). In a study investigating cell-mediated immune mechanisms in MS, a variant of GALC (rs2119704) showed a combined *p*-value of 2.20E-10 (*P*_*dis*_ = 3.50E-10, *P*_*rep*_ = 2.50E-02) (Sawcer et al., 2011).

The lack of GALC may result in neurodegeneration independent of inflammation (Sawcer et al., 2011). Scott-Hewitt et al. explored the mechanism underlying mutant GALC-associated neurodegeneration in animals model of demyelination, in which wild-type (GALC^+/+^) and GALC^±^ mice were exposed to cuprizone (Kipp et al., 2009). GALC^–/–^ mice were more defective compared to other genotypes (Scott-Hewitt et al., 2017). No significant behavioral or histological differences (e.g., in corpus callosal myelin) were observed between the two genotypes of animals at baseline. However, following demyelinating injury, GALC^±^ mice exhibited reduced remyelination and impaired myelin debris clearance, which might be caused by microglia dysfunction (Scott-Hewitt et al., 2017).

HLA-DO is a heterodimer formed by HLA-DOα and HLA-DOβ, encoded by HLA-DOA and HLA-DOB genes. HLA-DO is the human equivalent of murine H2-O, a non-classical MHC class II-like protein encoded in the class II region of the MHC (Trowsdale and Kelly, 1985). HLA-DO is only expressed in B cells and thymic epithelial cells (Douek and Altmann, 1997). B cells have been recognized as a contributor to the development of MS (Li et al., 2018). This study found that the HLA-DOB gene showed a twofold upregulated expression level in MS patients in contrast with healthy controls in PBMCs. To further verify the expression level of the HLA-DOB gene in B cells, we sorted B lymphocytes to compare the expression level of HLA-DOB in MS patients and healthy subjects. Moreover, the transcriptional level of the HLA-DOB gene was upregulated in MS patients in contrast with controls.

HLA-DO is strongly associated with HLA-DM and DO-DM complexes. It is a critical regulator in intracellular transport and essential for the survival in lysosomal MHC class II compartments surrounded by highly acidic B cells (Liljedahl et al., 1996; van Ham et al., 2000). Nagarajan et al. (2002) showed that HLA-DO regulated peptide loading and antigen presentation in B cells. Early findings showed that DO inhibited DM to prevent DM from removing the invariant chain peptide CLIP (Denzin et al., 1997). However, some studies reported that DO-KO mice did not show reduced MHCII-CLIP levels (Perraudeau et al., 2000; Brocke et al., 2003). Also, DO, together with DM, would allow a more fine-tuned epitope selection (Poluektov et al., 2013). The HLA-DOB gene has also been identified as a genetic variant in many diseases, such as KD (Shendre et al., 2014), type 1 diabetes mellitus (Qiu et al., 2015), and rheumatoid arthritis (Jiang et al., 2016). Further investigations are needed to explore HLA-DOB’s role in MS, especially in B cell antigen-presenting function.

The TNFRSF1A gene encodes TNFRSF1A protein tumor necrosis factor receptor 1 (TNFR1), which binds to tumor necrosis factor-alpha (TNFα) (Himmler et al., 1990), activating the transcription factor NF-κB and downstream pathways related to inflammation and apoptosis (Gregory et al., 2012). It has been demonstrated that TNFRSF1A (rs1800693, *p* = 1.59 × 10^–11^) and rs1800693 [T] allele are positively correlated with MS susceptibility (De Jager et al., 2009). The SNP rs1800693 in TNFRSF1A was identified in MS patients and control subjects (International Multiple Sclerosis Genetics Consortium [IMSGC], 2011). In a case-control cohort, rs1800693 showed a significant association with MS (*p*_*unc*_ = 0.011, *p*_*c*_ = 0.033) while rs4149584 did not (Hoffjan et al., 2015).

Increasing evidence suggests that TNFSF14 may be an MS susceptibility gene. TNFSF14, also known as LIGHT, was identified as one of the risk loci for MS (rs1077667^*G*^, *p* = 9.4 × 10^–14^) (Sawcer et al., 2011). It may regulate the function of T cells via microRNA (Jernås et al., 2013). Soluble LIGHT plays a protective role via inhibiting inflammation of the GG genotype of rs1077667. MS patients with this genotype showed the lowest LIGHT serum level (*p* = 0.02) compared to control subjects (Malmeström et al., 2013).

## Conclusion

In summary, in an MS GWAS dataset, we performed gene-based tests to get the intersection of MS risk genes, following the gene expression analysis that illustrated the gene characteristics in transcription level. Notably, the genes involved in antigen presentation and lysosomal function showed great differential expression in MS patients’ samples compared to controls. Furthermore, our finding provided support for these candidate genes in the participation of MS mechanisms.

## Data Availability Statement

Publicly available datasets were analyzed in this study. This data can be found here: www.ncbi.nlm.nih.gov/geo https://pubmed.ncbi.nlm.nih.gov/21833088/.

## Ethics Statement

The studies involving human participants were reviewed and approved by the Ethical Committee of Tianjin Neurological Institute. The patients/participants provided their written informed consent to participate in this study.

## Author Contributions

HL and WJ conceived and designed the study for MS. HL analyzed the GWAS data and wrote the manuscript. WJ was responsible for research supervision and manuscript revision. XH, YL, XZ, PC, and GX provided technical support. FX and XW provided suggestions for revision and proof of the manuscript. All authors approved the final version for submission.

## Conflict of Interest

The authors declare that the research was conducted in the absence of any commercial or financial relationships that could be construed as a potential conflict of interest.

## Footnotes

1 http://pngu.mgh.harvard.edu/purcell/plink/

2 www.ncbi.nlm.nih.gov/geo

## Abbreviations

CLIP: class II-associated invariant chain peptide
CNS: central nervous system
GAGs: glycosaminoglycans
GEO: gene expression omnibus
GWAS: large-scale genome-wide association studies
HCV: hepatitis C virus
IMSGC: International MS Genetics Consortium
KD: Krabbe disease
LD: linkage disequilibrium
MHC: major histocompatibility complex
MPSs: mucopolysaccharidoses
MS: multiple sclerosis
PBMC: peripheral blood mononuclear cells
PBTC: peripheral blood T cells
SNP: single nucleotide polymorphism
TNF α: tumor necrosis factor-alpha
VEGAS2: versatile gene-based association study-2 version 2.
